# Clinical Value of Pharmacogenomic Testing in a Patient Receiving FOLFIRINOX for Pancreatic Adenocarcinoma

**DOI:** 10.3389/fphar.2018.01309

**Published:** 2018-11-15

**Authors:** Lisa M. Velez-Velez, Caren L. Hughes, Pashtoon Murtaza Kasi

**Affiliations:** ^1^Department of Hematology and Oncology, Mayo Clinic, Jacksonville, FL, United States; ^2^Department of Pharmacy, Mayo Clinic, Jacksonville, FL, United States

**Keywords:** pharmacogenomics, *DPYD*, *UGT1A1*, *BRCA*, irinotecan, 5-fluorouracil, pancreas cancer, FOLFIRINOX

## Abstract

Pharmacogenomic testing may have clinical value in the treatment of patients with gastrointestinal malignancies such as colorectal and pancreatic cancer. These types of cancer are often treated with combination chemotherapy regimens. These regimens can lead to severe adverse effects in patients with diminished drug tolerability potentially due to certain genetic variants in the enzymes involved in the metabolism of the chemotherapies. Genetic variants resulting in decreased enzymatic activity of uridine diphosphate glucuronosyltransferase 1A1 (UGT1A1) and dihydropyrimidine dehydrogenase (DPD) are known to increase irinotecan and 5-fluorouracil-related toxicity, respectively. We report a case of a patient with pancreatic adenocarcinoma who was found to be not only homozygous for the *UGT1A1^∗^28* allele, but also heterozygous for a *DPYD* variant through pharmacogenomic testing. Potentially severe adverse effects were prevented in this patient’s case by implementing preemptive dose reductions. On the basis of the significant implications of chemotherapy-related toxicity in this and other similar cases, we report on the clinical value of integrating pharmacogenomic testing into clinical practice to allow for preemptive and/or point-of-care dose reductions in patients potentially at risk for increased toxicity. This is even more important in an era where combinatorial triplet chemotherapies are increasingly being used.

## Introduction

Pharmacogenomic testing can have significant implications for patients with gastrointestinal cancers by reducing toxicity in combination chemotherapy regimens. Combination regimens with 5-fluorouracil (5-FU), leucovorin, irinotecan, and oxaliplatin, such as FOLFIRINOX for pancreatic cancer or FOLFOXIRI for colorectal cancer, are standard of care being used by many clinicians for patients with advanced or metastatic pancreatic or colorectal cancer with good performance status (Balaban et al., 2016; Leal et al., 2017). Treatment with irinotecan and 5-FU as single agents or in combination may result in severe adverse effects including severe diarrhea, mucositis, and neutropenia. In the TRIBE trial, the FOLFOXIRI arm was significantly associated with higher incidence of grade 3 or 4 neutropenia (50% vs. 20.5%), diarrhea (18.8% vs. 10.6%), and stomatitis (8.8% vs. 4.3%) when compared with the FOLFIRI plus bevacizumab group (Loupakis et al., 2014). Similarly, when compared with gemcitabine alone, FOLFORINOX treatment was associated with significantly higher incidences of grade 3 or 4 neutropenia (45.7% vs. 21%), febrile neutropenia (5.4% vs. 1.2%), diarrhea (12.7% vs. 1.8%), and thrombocytopenia (9.1% vs. 3.6%) (Conroy et al., 2011).

The metabolism of irinotecan involves the inactivation of the active metabolite SN-38 to the pharmacologically inactive SN-38 glucuronide by the uridine diphosphate glucuronosyltransferase 1A1 (UGT1A1) enzyme in the liver (Iyer et al., 1998). *UGT1A1^∗^28* is a known polymorphism in which there is a (TA)_7_TAA sequence in the promoter region of *UGT1A1* instead of (TA)_6_TAA. It has been shown that homozygous and heterozygous patients for *UGT1A1^∗^28* exhibit more severe grades of diarrhea and neutropenia as a result of irinotecan-induced toxicity (Iyer et al., 2002; Liu et al., 2014). Irinotecan is often used in combination with 5-FU, a fluoropyrimidine that is also associated with similar adverse effects. Following administration, more than 80% of 5-FU is inactivated by the enzyme dihydropyrimidine dehydrogenase (DPD) in the liver (Diasio and Harris, 1989). The activity of DPD has been shown to be reduced in four single nucleotide polymorphisms in *DPYD* – c.1905 + 1G > A (rs3918290, DPYD^∗^2A), c. 1679T > G (rs55886062, DPYD^∗^13), c. 2846A > T (rs67376798), and c.1129-5923C > A (rs75017182, c.1236G > A/HapB3) – which have proved to be relevant clinical predictors of fluoropyrimidine-associated toxicity (Meulendijks et al., 2015).

Although the toxicity-associated effects of decreased enzymatic function caused by the *UGT1A1* and *DPYD* variants have been widely studied, few guidelines have been developed to assist physicians in pharmacogenomic-based dosing of irinotecan, 5-FU, and its oral pro-drug capecitabine in patients at risk (Hoskins et al., 2007; Ruzzo et al., 2017). Furthermore, there is no consensus on recommendation of upfront *UGT1A1* and *DPYD* genotyping as part of cancer treatment protocols. Despite accumulating evidence that upfront *DPYD* and *UGT1A1* genotyping is feasible and may improve safety of irinotecan and fluoropyrimidine therapy for patients, guidelines for universal pretreatment genotyping in clinical practice have not been established in the United States and so it is not supported by the National Comprehensive Cancer Network Panel at present (Deenen et al., 2016; Cremolini et al., 2017).

Pharmacogenomic tests are commercially available and may be performed prior to starting chemotherapy. These can be ordered as single gene and/or part of pharmacogenomics panel tests. The test results may help physicians make dosing adjustments in the treatment regimens to increase tolerability. Variability in the few published pharmacogenomics-based dosing guidelines and lack of knowledge about its feasibility and benefits might be reasons why preemptive pharmacogenomic testing has not become common practice.

Herein, we report a case of a patient with pancreatic adenocarcinoma who was found to be a carrier of deleterious polymorphisms in both *UGT1A1* and *DPYD* during our recently implemented preemptive and/or point-of-care pharmacogenomics-based genetic testing platform, placing him at increased risk for chemotherapy-related toxicity.

With respect to preemptive pharmacogenomics testing, at our institution, the two genes can be individually tested through blood tests run at the Mayo Clinic laboratories and/or through our Center of Individualized Medicine (CIM) platform employing the commercially available OneOme RightMed pharmacogenomic comprehensive test, which reports on 23 genes of interest. The feasibility of this, alongside our pilot studies, was reported at the American Society of Clinical Oncology (ASCO) meeting in June 2018 (details noted in section “Discussion”).

## Case Presentation

A 47-year-old man presented to his local primary care physician with symptoms of diarrhea and clay-colored stools in April 2018. Laboratory studies revealed direct hyperbilirubinemia which prompted further workup. A right upper quadrant ultrasound showed gallbladder sludge and dilation of the common bile duct to 12 mm indicating obstructive jaundice. No intrahepatic ductal dilatation was appreciated. Computed tomography scan of the abdomen showed a mass in the head of the pancreas which was found to measure approximately 2.6 cm × 1.4 cm in subsequent magnetic resonance imaging (MRI) examination. No evidence of metastasis was found. The patient then underwent an endoscopic retrograde cholangiopancreatography and placement of a plastic biliary stent across a focal stricture of the distal common bile duct secondary to the pancreatic head mass. A diagnosis of pancreatic adenocarcinoma was made.

Upon transfer to our institution in May 2018, laboratory workup revealed hyperbilirubinemia and elevated liver enzymes. In response to the hepatic workup abnormalities, we started the patient on a neoadjuvant regimen of FOLFIRINOX with reduced doses of 5-FU and irinotecan plus standard doses of oxaliplatin and leucovorin. In cycle 1, the standard dose of irinotecan (180 mg/m^2^) was reduced by 50% and 5-FU was only administered as a bolus due to lack of port in the patient in the first cycle and the hyperbilirubinemia. The patient received full dose of oxaliplatin (85 mg/m^2^ = 190 mg) and leucovorin (400 mg/m^2^ = 900 mg) along with the reduced dose of irinotecan (90 mg/m^2^ = 200 mg) and the 5-FU bolus (400 mg/m^2^ = 900 mg) on day 1 (Table 1). The dosing adjustments were implemented to prevent potentially serious toxicity-related adverse effects from 5-FU and irinotecan while we awaited the results from the *UGT1A1* and *DPYD* genotyping tests, which we have recently integrated into our clinical practice as a quality improvement initiative. We planned to escalate the chemotherapy regimen to full dose for cycle 2 depending on the results from the genetic tests. Despite the marked dose reductions in cycle 1, blood tests prior to initiation of cycle 2 revealed grade 2 neutropenia with an absolute neutrophil count of 1.17 × 10^9^/L.

**Table 1:** Dosing adjustments implemented in the FOLFIRINOX regimen.

Drug (Standard dose)	Cycle 1	Cycle 2	Cycle 3
Irinotecan	200 mg	200 mg	200 mg
(180 mg/m^2^)	(50% reduction)	(50% reduction)	(50% reduction)
5-FU – Bolus	900 mg	Not administered	450 mg
(400 mg/m^2^)			(50% reduction)
5-FU – Continuous infusion over 2 days	Not administered	1350 mg	2700 mg
(2400 mg/m^2^)		(75% reduction)	(50% reduction)
Leucovorin	900 mg	900 mg	900 mg
(400 mg/m^2^)			
Oxaliplatin	190 mg	190 mg	190 mg
(85 mg/m^2^)			


Prior to administration of cycle 2 of the dose-adjusted FOLFIRINOX, we received the pharmacogenomic test results, which indicated that the patient is homozygous for the *UGT1A1^∗^28* allele and an heterozygous carrier of a *DPYD* variant identified as c.536dupC, a no-activity allele. The pharmacogenomics findings in our patient guided us to maintain some of the dose reductions alongside further modifications based on pharmacogenomics e-consult. Additionally, given the neutropenia, growth factor support was also added to maintain the curative-intent intensity of neoadjuvant therapy. Adjustments in cycle 2 consisted of the same 50% reduction in the dose of irinotecan and a 75% reduction in the 5-FU dose (600 mg/m^2^ = 1350 mg administered over 2 days from day 1 to day 2) plus omission of the 5-FU bolus. The patient continued receiving standard doses of oxaliplatin and leucovorin in both cycle 2 and 3. Dosing adjustments from cycle 2 were maintained in cycle 3, except for 5-FU which was given as the bolus dose reduced by only 50% (200 mg/m^2^ = 450 mg on day 1) and the continuous infusion was escalated to 50% (1200 mg/m^2^ = 2700 mg administered over 2 days) along with pegfilgrastim support starting in cycle 2. No other dosing modifications or escalations were done in cycles 3–5. There was one admission to the ER for diarrhea during cycle 3 requiring IV fluids. This did not occur in cycles 4 and 5.

Because of the young age of onset of pancreatic adenocarcinoma in the patient and a maternal history of breast cancer with a confirmed *RAD51C* variant (c.790G > A), we performed comprehensive testing to evaluate the following genes: *APC, ATM, BMPR1A, BRCA1, BRCA2, BRIP1, CDH1, CDK4, CDKN2A (*p14ARF*), CDKN2A* (p16INK4a), *CHEK2, EPCAM, FANCC, MEN1, MLH1, MSH2, MSH6, NBN, NF1, PALB2, PALLD, PMS2, PTEN, RAD51C, RAD51D, SMAD4, STK11, TP53, TSC1, TSC2*, and *VHL*. Results were negative for all genes that were evaluated.

**FIGURE 1:**
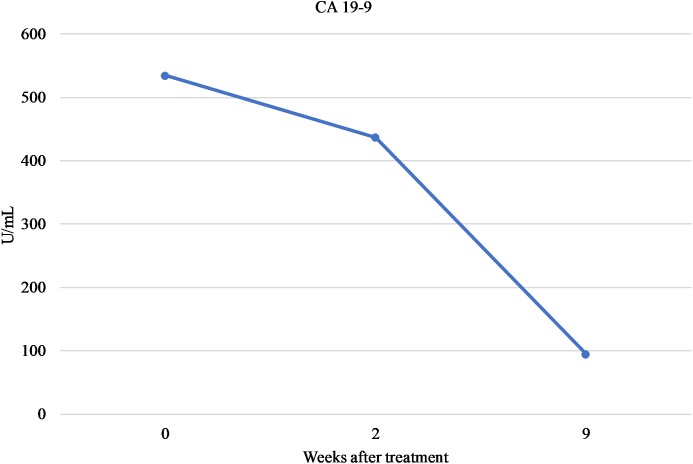
Serum levels of CA 19-9.

To date, the patient initially received 5 cycles of neodjuvant FOLFIRINOX. The treatment had been well-tolerated with no evidence of severe adverse effects. At the most recent follow-up, MRI examination showed reduction in the size of the tumor to approximately 1.9 cm × 1.6 cm. Furthermore, serum levels of CA 19-9 have steadily declined (Figure 1). Patient subsequent had the disease resected (R0 resection) and is completing the remainder of the cycles adjuvantly with a plan for a total of 6 months of chemotherapy (12 cycles). Clinical course so far indicates both response to the treatment and tolerability despite the deleterious polymorphisms in *UGT1A1* and *DPYD* and the implemented dose adjustments.

## Discussion

Deficiencies in enzymatic function of UGT1A1 and DPD caused by genetic polymorphisms may result in decreased metabolism of irinotecan and 5-FU, respectively, leading to increased risk for chemotherapy-induced toxicity. A dose-finding and pharmacokinetic study supports a role for genotype-directed dosing of irinotecan without compromising antitumor efficacy in patients receiving lower doses due to their *UGT1A1^∗^28* homozygous genotype (Innocenti et al., 2014). Similarly, evidence from a meta-analysis of several prospective and retrospective cohort studies supports a role for upfront screening for the clinically validated *DPYD* variants (*DPYD^∗^2A*, c.2846A > T, c.1679T > G, and c.1236G > A/HapB3) followed by dose adjustment to increase safety in variant carriers treated with fluoropyrimidines (Meulendijks et al., 2015). This need for dose modifications of irinotecan and 5-FU in patients with reduced UGT1A1 and/or DPD activity is recognized by the U.S. Food and Drug Administration, but no specific dose adjustments are recommended in the drug labels on the basis of insufficient data.

Currently, published guidelines to assist clinicians in linking pharmacogenomic testing results to appropriate adjustments in chemotherapy dosing are limited, hence the differences among dosing modifications that have been reported in the literature (Tables 2, 3). Available published recommendations based on systematic literature reviews include guidelines developed by the Clinical Pharmacogenetics Implementation Consortium (CPIC) for *DPYD* genotyping and fluoropyrimidine dosing and by the Pharmacogenetics Working Group of the Royal Dutch Association for the Advancement of Pharmacy for both *UGT1A1* and *DPYD* genotyping and drug dosing (Swen et al., 2011; Amstutz et al., 2018). Another set of professional guidelines for irinotecan therapy in patients carrying the deficient *UGT1A1^∗^28* allele was developed by the French Group of Clinical Onco-Pharmacology and the National Pharmacogenetics Network (Etienne-Grimaldi et al., 2015). This group also developed guidelines to recommend *UGT1A1* genotyping in patients who are going to receive irinotecan doses of ≥180 mg/m^2^. Although these guidelines provide information that allows clinicians and pharmacists to interpret the implications of pharmacogenomic testing results, there is considerable heterogeneity among their recommendations.

**Table 2:** Pharmacogenomics-based recommendations for irinotecan dosing adjustments.

Study	Dose recommendation for heterozygous patients *(^∗^1/^∗^28*)	Dose recommendation for homozygous patients *(^∗^28/^∗^28*)
Swen et al., 2011	Dose reduction is not recommended.	If dose >250 mg/m^2^: reduce by 30%, then increase dose according to neutrophil count.
		If dose ≤250 mg/m^2^: no dose adjustment.
Etienne-Grimaldi et al., 2015	Rigorous clinical surveillance is recommended.	If dose 180–230 mg/m^2^: 25–30% reduction in the first cycle.
		Dose ≥240 mg/m^2^: not recommended.
Innocenti et al., 2014	Patients received irinotecan as a single agent every 3 weeks. Patients with the *^∗^1/^∗^28* genotype tolerated an increased dose (390 mg/m^2^).	A 20% dose reduction from the standard 350 mg/m^2^ was not safe. Instead, a 40% dose reduction to 220 mg/m^2^ seemed to be tolerated.
Miyata et al., 2016	No recommendation is offered.	Upfront dose reduction of 20% is recommended from the standard dose of irinotecan in Japan (150 mg/m^2^). Recommended reduction also applies to homozygous carriers of *UGT1A1^∗^6*, which is relatively common in Asians.


**Table 3:** Pharmacogenomics-based recommendations for fluoropyrimidine (i.e., 5-fluorouracil, capecitabine) dosing adjustments.

Study	*DPYD* genotype	Dose recommendation/adjustment
Swen et al., 2011	PM: An individual carrying two no function alleles or an individual carrying one no function plus one decreased function allele.	PM: Avoid use of 5-FU or capecitabine.
		IM: Select alternative drug or upfront dose reduction of 50%, then increase dose according to toxicity and efficacy.
Amstutz et al., 2018 (CPIC guidelines)	IM: An individual carrying one normal function allele plus one no function allele or one decreased function allele, or an individual carrying two decreased function alleles.	PM: Avoid use of 5-FU, or reduce starting dose by >75% and do early therapeutic drug monitoring.
		IM: Reduce starting dose by 25–50%, then adjust dose based on toxicity.
Meulendijks et al., 2015	Heterozygous for c.1679T > G	Upfront 50% reduction.
	Heterozygous for c.1236G > A/HapB3	Upfront 25% reduction.
Meulendijks et al., 2015, 2016	Homozygous for c.1236G > A/HapB3	Upfront 50% reduction.
Henricks et al., 2018	Novel *DPYD* genotype (i.e., amplification of exons 17 and 18 of *DPYD* and heterozygosity for *DPYD^∗^2A*)	Capecitabine dose reduced to 77 mg/m^2^ once every 5 days (0.8% of original dose). Pharmacokinetic analyses showed adequate drug exposure despite dose reduction.
Deenen et al., 2016	*DPYD^∗^2A*	Upfront 50% reduction is safe and adequate 5-FU systemic exposure is maintained.


*PM, poor metabolizer; IM, intermediate metabolizer; CPIC, Clinical Pharmacogenetics Implementation Consortium.*

The importance of research evaluating the feasibility and clinical value of preemptive pharmacogenomic testing is being increasingly recognized. A multicenter clinical trial is currently assessing the impact of pretreatment pharmacogenomic testing and dosing modifications on the incidence of adverse events in patients set to receive one or more of the 42 drugs for which a Dutch Pharmacogenetics Working Group guideline is available (ClinicalTrials.gov, 2017b; identifier NCT03093818). Another study in an oncology clinic is also assessing the feasibility of implementing preemptive pharmacogenomics testing for colorectal cancer patients (ClinicalTrials.gov, 2017a; identifier NCT03187184). Interestingly, genotype-guided dosing of irinotecan is also being studied in the context of potential benefit from dose escalations in patients with *UGT1A1^∗^1/^∗^1* and *^∗^1/^∗^28* genotypes as the approach has already been successfully accomplished in early-phase trials (Toffoli et al., 2010, 2017). Some of these dose-escalation studies are being conducted in patients receiving modified FOLFIRINOX for advanced gastrointestinal malignancies (ClinicalTrials.gov, 2018b; identifier NCT01643499) and FOLFIRI plus bevacizumab for metastatic colorectal cancer (ClinicalTrials.gov, 2018a; identifier: NCT02138617).

Preliminary studies in our practice suggest that pharmacogenomic testing to enable genotype-driven dosing of irinotecan and 5-FU is a feasible strategy to help prevent potentially severe adverse effects from chemotherapy. As exemplified by the case reported here, we have found a higher prevalence of *UGT1A1* and *DPYD* variants in our patient cohort than what is reported in the literature (Baldeo et al., 2018a). This finding along with our clinical experience with a life-threatening case of 5-FU toxicity prompted us to implement a pharmacogenomics-based genetic testing platform in our practice (Baldeo et al., 2018b). This pharmacogenomics platform has enabled us to better manage treatment regimens in patients at increased risk through patient-specific medication management consults with pharmacists in our institution (Hughes et al., 2018). Increased tolerability has been observed in our patients while maintaining treatment efficacy. Detailed analyses on these cohorts of patients as part of a quality improvement project with our center of individualized medicine are ongoing.

## Concluding Remarks

*UGT1A1* and *DPYD* genotyping are important tools available today to identify patients at increased risk of chemotherapy-related toxicity prior to starting irinotecan and fluorouracil-based regimens. Our case describes a patient who has been successfully treated with FOLFIRINOX despite having decreased enzymatic activity of both UGT1A1 and DPD and thus being at increased risk for chemotherapy-induced toxicity. With quicker turnaround and reduced costs of these pharmacogenomic tests, our group has shown that integrating preemptive and/or point-of-care usage of this is feasible.

## Informed Consent

Written informed consent was obtained from the patients for their anonymized information to be included in this report.

## Author Contributions

LV-V wrote the manuscript. CH contributed to editing and approval of the final manuscript. PK contributed the patient data, guided the initial draft, edited, and approved the final manuscript.

## Conflict of Interest Statement

The authors declare that the research was conducted in the absence of any commercial or financial relationships that could be construed as a potential conflict of interest.
